# Short-Term Analysis (8 Weeks) of Social Distancing and Isolation on Mental Health and Physical Activity Behavior During COVID-19

**DOI:** 10.3389/fpsyg.2021.652086

**Published:** 2021-03-18

**Authors:** Jessica Ann Peterson, Grant Chesbro, Rebecca Larson, Daniel Larson, Christopher D. Black

**Affiliations:** ^1^Sensory and Muscle Function Laboratory, Department of Health and Exercise Science, University of Oklahoma, Norman, OK, United States; ^2^Body Composition and Human Performance Laboratory, Department of Health and Exercise Science, University of Oklahoma, Norman, OK, United States; ^3^Sports Performance and Sports Business Analytics, Department of Health and Exercise Science, University of Oklahoma, Norman, OK, United States

**Keywords:** coronavirus, depression, anxiety, mood, loneliness, exercise

## Abstract

Due to the COVID-19 pandemic, cities and states adopted social distancing, social isolation, or quarantine measurements to slow the transmission of the disease. Negative mental health outcomes including depression and anxiety have been associated with social distancing or social isolation. The purpose of the present study was to examine changes in psychological health and physical activity over an 8 week period under social distancing policies during the COVID-19 pandemic.

**Methods:** Ninety (73.3% female; age 32.04 ± 11.33) individuals participated in this study. Qualifying participants answered questions using an online survey regarding their loneliness, depressive symptoms, anxiety symptoms, mood state, and physical activity over four time points each lasting two weeks.

**Results:** Symptoms of depression and state anxiety were increased in the population when compared to nationwide statistics from before the COVID-19 pandemic. Time point 2, ~1 month into social isolation, showed the most significant effects on mental health. During this time point, 100% of the participants showed symptoms of depression. There were no significant changes in physical activity over the 8 weeks. Loneliness, depressive symptoms, fatigue, and mood state were negatively associated with participation in physical activity. Vigor and state anxiety were associated with participation in physical activity.

**Conclusion:** Social isolation and social distancing practices have had a negative effect on depression, anxiety, and mood over time. It appeared that depressive symptoms and total mood disturbance was elevated during time point two. Depressive symptoms were much higher than average compared to previous epidemiological data. Physical activity amount did not change over time but was associated with poor mental health.

## Introduction

State and local governments in the United States introduced social distancing policies that began early spring of 2020 in response to the novel coronavirus disease (COVID-19) pandemic. To mitigate the spread of the disease; shelter in place and stay at home orders were mandated leading to lifestyle modifications. These orders encouraged individuals to work from home, utilize telecommunication methods, and reduce activities outside the home to essential errands. To slow the spread of the virus, self-quarantine, social isolation, and social distancing were encouraged and included closing of places of worship and businesses including gyms and restaurants. While social distancing and stay at home orders were essential for slowing transmission of the virus, it increased the risk that these interventions could have detrimental effects on physical and psychological health.

Given the situation of safer at home practices and social isolation, special attention should be paid to mental health. Depression, stress, and anxiety have been shown to be significant burdens on society during the COVID-19 pandemic (Castelli et al., 2020; Huang and Zhao, 2020; Rajkumar, 2020; Sønderskov et al., 2020; Wang et al., 2020). It has been suggested that social distancing and self-quarantine may have contributed to these negative mental health states (Xiao et al., 2019). Individuals undergoing social isolation or social distancing can have unpleasant experiences including loneliness, detachment from relationships, uncertainty about the future, boredom, and loss of freedom (Brooks et al., 2020). Loneliness has been defined as the embodiment of social isolation and can show an individual's negative feelings about the frequency and closeness of their social contacts (Steptoe et al., 2013). In addition, loneliness has been associated with increased levels of depressive symptoms (Cacioppo et al., 2006; Matthews et al., 2016; Palgi et al., 2020; Rosenberg et al., 2020), increased symptoms of anxiety (Ernst and Cacioppo, 1999; Cacioppo et al., 2006; Okruszek et al., 2020; Palgi et al., 2020), and an altered mood state (Loucks, 1980; Besser et al., 2020). Prolonged time at home, loneliness (Page and Hammermeister, 1995; Hawkley et al., 2009; Richard et al., 2017; Schrempft et al., 2019; Creese et al., 2020), and mental health disturbances (Mayou et al., 2000; Brummett et al., 2003; Stewart et al., 2003; Van Gool et al., 2003; Allan et al., 2007; Da Silva et al., 2012; Legey et al., 2017; Stubbs et al., 2017; Creese et al., 2020; López-Bueno et al., 2020; Stanton et al., 2020) can increase behaviors that promote sedentary behavior and decrease overall physical activity (Biddle, 2016; Creese et al., 2020).

Insufficient physical activity has been shown to be a key risk factor for negative psychological and physical health including increased risks for cardiovascular, pulmonary, and metabolic diseases (Warburton et al., 2006). Individuals are recommended to participate in at least 150 min of moderate-intensity physical activity, 75 min of vigorous-intensity physical activity, or some combination of the two during a week (Organization, 2020). However, recent recommendations suggest that, during confinement, individuals should increase physical activity amount beyond than these recommendations to compensate for the increase in sedentary time at home (Jiménez-Pavón et al., 2020). It has been shown that current physical activity adherence to WHO guidelines during the initial phase of confinement due to COVID-19 lowered perceived anxiety and improved mood (López-Bueno et al., 2020) and that a reduction of total physical activity had a negative impact on psychological health (Maugeri et al., 2020). Closure of gyms in Oklahoma when stay at home orders were enacted meant fewer opportunities to partake in physical activity. However, there was no restriction on outdoor activity when compared to other western countries; the United Kingdom for example, was limited to one hour of outdoor activity with members of the same household, once per day (gov.uk., 2020).

The ambiguity surrounding COVID-19 presents a unique opportunity to examine physical activity behaviors and mental health over time. Research to date has shown that social distancing or social isolation can reduce physical activity (Maugeri et al., 2020) and psychological well-being and can increase depressive symptoms (Mazza et al., 2020; Sønderskov et al., 2020; Wang et al., 2020), anxiety symptoms (Cao et al., 2020; Mazza et al., 2020; Sønderskov et al., 2020; Wang et al., 2020), and feelings of psychological distress (Qiu et al., 2020; Wang et al., 2020) during the COVID-19 pandemic. Limited research has examined psychological health and physical activity levels longitudinally during periods of loneliness especially in younger populations. In view of this context, it was necessary to examine the changes in mental health and physical activity behaviors associated with social distance practices throughout the implemented duration of “safer at home orders” in place in Oklahoma. Therefore, the purpose of this study was to examine changes in psychological health and physical activity over an 8 week period under social distancing policies in response to the COVID-19 pandemic. Based upon the research to date, we hypothesized that time would have an impact on mental health outcomes and physical activity amount, in that a longer time at home would have a negative effect on mental health and lead to reduced physical activity amount.

## Materials and Methods

### Sample

A total of 304 participants completed the first questionnaire (2–4 weeks following stay at home orders were in place), however the final sample included 199 participants that remained in the study due to incomplete data. These 199 participants were invited back for round two of data collection. During time point 2 (4–6 weeks), a total of 156 responses were gathered. However, during time point 3 (6–8 weeks) only 118 participants returned the survey. The final time point which was taken when stay at home orders in Oklahoma were relaxed, 100 people responded to the questionnaire. After filtering out the data by checking for incompletes and inconsistencies; 90 people were included in the analysis. Using G^*^Power 3.1.9.2, a computed a priori sample size of 82 was the required sample size for a repeated measures, between factors ANOVA with an approximate effect size of 0.25, alpha of 0.05, and estimated power of 0.8, with four time points. Informed consent was gathered using yes/no prompt following written instructions on how the data will be gathered, used, and protected before the questionnaire was presented to the participants. All instruments were approved by the University of Oklahoma ethics committee and complied with the Declaration of Helsinki. Our sample inclusion criteria was that participants had to be between the ages of 18–64 (73.3% of which were female), could read English, and lived in an area were shelter in place policies were enforced to encourage social distancing. No further exclusion criteria were imposed.

### Procedure

Participants were recruited using the following recruitment strategies; (1) campus wide emails, (2) social media posts, (3) snowballing family and friend recruitment of people who had completed the questionnaire. Participants were invited to click a link to complete the questionnaire using Qualtrics software (Qualtrics, XM, Provo, UT). Participants were informed that they would receive a follow up email every 2 weeks with a new link to the same questionnaire to be completed, if they remained in the study. The initial email was sent with the Qualtrics link, 2 weeks after the initial stay at home orders were in place; this survey was open for 2 weeks before closing at 4 weeks (04/06/2020–04/20/2020) and participants were asked to recollect information from the previous week (03/30/2020–04/13/2020) regarding demographic data, self-reported physical activity from the previous 7 days, a depression scale, a loneliness scale, questions regarding mood state, and finally questions addressing situational and trait anxiety. The second email was sent only to participants that had completed the initial questionnaire and data was collected between 04/20/2020 until 05/04/2020, again with participants being asked to report their physical activity, and psychological wellbeing from the previous week (04/13/2020–04/27/2020). The third-time point included participants that had remained in the study until this point and included weeks 4–6 of social distancing orders (05/04/2020–05/18/2020), recollecting data from the previous week (04/27/2020–05/11/2020). The final time point was collected after phase 1 had begun in the state of Oklahoma (May 1st) and approximately when phase 2 had begun (May 15th) which included the reopening of social environments such as bars, summer camps, and organized sporting events.

### Instruments

Questionnaires were used to collect data on demographics, loneliness, depressive symptoms, state and trait anxiety, mood, and self-report physical activity.

Demographics were collected and participants were asked to self-report their sex, year that they were born, education level, what state they lived in, their current employment status, how many people they currently lived with, and their living situation; whether they owned, rented, or lived with their parents.

The UCLA Loneliness Scale (UCLA-LS) measures a person's subjective feelings of general loneliness and feelings of social isolation over 20 items (Russell, 1996). Individuals rate each item based upon how they feel on a four point scale from often to never. The range of possible scores is 0–60, with higher scores signifying greater loneliness.

The Center for Epidemiological Studies Depression Scale **(**CES-D) is used to measure cognitive and affective depressive symptomology (Radloff, 1977). This scale examines depressed mood; feelings of guilt and worthlessness; feelings of helplessness and hopelessness; psychomotor retardation; loss of appetite; and sleep disturbance. Scoring is between 0 and 3 with 0 indicating that the participant felt this way rarely or none of the time and three indicating that the participant felt this way most or all of the time. Possible ranges of scores run from 0 to 60, with higher scores indicating the presence of more depressive symptomatology. The CES-D scale has been validated with having very good internal consistency showing an alpha of 0.85 in general populations

The State-Trait Anxiety Inventory (STAI) Form Y measures current or situational anxiety (Spielberger, 2010). The S-anxiety scale (State Anxiety; STAI Form Y-1) consists of twenty statements that evaluate how respondents feel “right now, at this moment.” The T-Anxiety scale (Trait Anxiety; STAI Form Y-2) consists of twenty statements that assess how people generally. Inventory items are given a weighted score on a five point intensity scale with 0 being “not at all” to 4 being “extremely.” Anxiety scores can vary from a minimum of 20 to a maximum score of 80. A higher score in both trait and state scales indicates the presence of more anxiety related symptomology.

The modified Profile of Mood States (POMs), a shortened version of a validated psychological test regarding mood (Grove and Prapavessis, 1992), was used to assess six different mood states over the previous 7 days; energy (vigor), fatigue, tension, depression, anger, and confusion. Subjects self-report on each of these areas using a 5-point Likert scale with 0 being not at all and 4 being extremely. A total mood disturbance (TMD) score was calculated by summing the totals for the tension, depression, fatigue, confusion, anger, and then subtracting the total for vigor as directed from original authors. A higher TMD score indicates a greater mood disturbance.

The International Physical Activity Questionnaires (IPAQ) measures self-reported physical activity over five activity domains asked independently (work-related physical activity, home-related physical activity, and leisure-related physical activity, time-spent sitting, and time-spent traveling). The participants are asked to recall and respond to open-ended questions regarding their physical activity from the past 7 days. Participants were asked the number of days that they did moderate, vigorous, or walking for each of the domains, and then how many minutes they performed that activity for including sitting and travel time. Metabolic equivalents (MET mins) for each exercise intensity (walking, moderate, vigorous) was calculated by calculating number of minutes multiplied by the number of days on which that activity was performed and adding each of the domains together of the same exercise intensity. The more MET-mins completed, the more active the individual was that week.

To examine participation in physical activity the participants were classified into three categories: low, moderate, and high (de Moraes et al., 2013). Moderate activity was defined as five or more days per week with a combination of walking, moderate intensity, and vigorous intensity activity totaling 600 MET mins/week. High activity was defined as 7 days with a combination of walking, moderated intensity, and vigorous intensity activity totaling 3,000 MET mins/week. Low activity was defined as not meeting the recommendations for either the moderate or high categories. In addition, the participants were further separated into two groups: sufficient and insufficient activity (de Moraes et al., 2013). The sufficient group consisted of those meeting the criteria for moderate or high levels of physical activity. And the insufficient group consisted of those in the low category.

### Statistical Analysis

Repeated measures ANOVA's were performed for all of the psychological mood variables (loneliness, anxiety symptoms, depressive symptoms, and mood state) and the physical activity variables (walking, moderate intensity physical activity, vigorous intensity physical activity, and total physical activity) to identify the effect of time over the 8 week time block. Normality was assessed using a Shapiro-Wilk test. A Bonferroni adjustment was used to identify differences in the variables over the time points. To examine relationships between the psychological measures and the physical activity measures, Pearson's r correlations were performed. All data were analyzed using SPSS version 24.0 (IBM Corp., Armonk, NY, USA). The significance level for all tests was set at *p* < 0.05.

## Results

A Shapiro-Wilk test was performed and the data were found to be normally distributed (*p* < 0.05). Demographic data is presented in Table 1.

**Table 1:** Demographic characteristics.

		**Number (*N*)**	**Percentage (%)**
**Age**			
	18–29	52	57.7
	30–39	24	26.7
	40+	14	15.6
**Sex**			
	Male	23	25.6
	Female	66	73.3
	Other	1	1.1
**Race**			
	White/Caucasian	74	82.2
	Black/African-American	3	3.3
	Native American	4	4.4
	Other	9	10
**Education**			
	< Bachelor's degree	26	28.9
	Bachelor's degree	31	34.4
	Graduate/professional degree	33	36.7
**Employment Status**			
	Working	68	75.5
	Not working (laid off)	7	7.8
	Not working (looking)	6	6.7
	Other	9	10
**Number of individuals in household**			
	1	31	34.4
	2	33	36.7
	3	16	17.8
	4+	10	11.1
**Living situation**			
	Own	42	46.7
	Rent	33	36.7
	Live with parent(s)	15	16.6

### Loneliness

A repeated measure ANOVA was performed using a Bonferroni adjustment between all four time points to identify changes in loneliness during the 8-week time block (Table 2). Mauchly's Test of Sphericity indicated that the assumption of sphericity was violated [χ^2^(5) = 23.44, *p* < 0.001], and therefore a Greenhouse-Geisser correction was used. There was no significant effect of time on loneliness [*F*_(2.603, 231.689)_ = 2.083, *p* > 0.05, η^2^ = 0.023]. There were no differences at any time point for loneliness.

**Table 2:** Descriptive Statistics examining differences in mental health outcomes and physical activity amount over four time points.

****	**Time Point 1**	**Time Point 2**	**Time Point 3**	**Time Point 4**
Loneliness	20.33 ± 14.32	20.39 ± 14.50	19.71 ± 16.38	17.62 ± 15.80
Depression	17.31 ± 10.53^***^	26.33 ± 3.86^*^, ^**^, ^***^	16.11 ± 11.94^***^	14.27 ± 10.86
State Anxiety	42.54 ± 4.71^***^	43.66 ± 5.01	42.73 ± 5.38	43.98 ± 5.23
Trait Anxiety	45.92 ± 4.38	45.64 ± 4.67	45.22 ± 5.02	44.86 ± 4.45
Anger	9.52 ± 3.53	9.81 ± 3.33	9.76 ± 4.09	8.98 ± 4.12
Vigor	12.40 ± 3.84	10.71 ± 3.12^*^, ^**^, ^***^	12.32 ± 4.52	12.99 ± 4.16
Fatigue	12.41 ± 4.78	13.08 ± 5.46^***^	12.74 ± 5.18^***^	11.47 ± 5.07
Confusion	10.06 ± 2.50	9.90 ± 3.2	9.59 ± 2.65	9.33 ± 2.85
TMD	41.05 ± 18.24^***^	43.26 ± 18.31^***^	39.7 ± 19.71^***^	35.91 ± 19.80
Walking (MET.mins/wk)	367.34 ± 366.07	363.49 ± 440.30	364.56 ± 639.21	399.49 ± 661.35
Moderate Intensity PA (MET.mins/wk)	399.50 ± 432.26	450.92 ± 633.90	478.94 ± 610.96	454.79 ± 525.46
Vigorous Intensity PA (MET.mins/wk)	185.70 ± 226.32	228.17 ± 343.47	214.61 ± 369.76	240.39 ± 411.63
Total PA (MET.mins/wk)	926.54 ± 840.68	1042.58 ± 1083.02	1058.11 ± 1305.24	1094.67 ± 1227.72

* *Difference compared to time point 1*;

** *Difference compared to time point 3*;

*** *Difference compared to time point 4; TMD, total mood disturbance; PA, physical activity*.

### Depression

Using a Greenhouse-Geisser correction [χ^2^(5) = 54.87, *p* < 0.001], there was a significant effect of time on depressive symptoms [*F*_(2.160, 192.240)_ = 60.916, *p* < 0.001, partial η^2^ = 0.41]. Time point 2 had the highest depression scores (26.33 ± 3.86) compared to time point 1 (17.31 ± 10.53, *p* < 0.001), time point 3 (16.11 ± 11.94, *p* < 0.001), and time point 4 (14.27 ± 10.86, *p* < 0.001) (see Table 2). In addition, time point 4 had lower depression scores than time point 1 (*p* < 0.01) and time point 3 (*p* < 0.001).

To determine if participants had symptoms of depression a cut-off score of 16 on the CES-D was used (Brummett et al., 2003). The highest rate of depression symptoms in the sample occurred at time point 2 (100%, 90/90). At time point 1, 50% (45/90) of the sample population met the criteria for symptoms of depression. A drop to 47% (42/90) occurred at time point 3 and a further drop to 40% (36/90) occurred at time point 4 which had the lowest rate of depressive symptoms.

### Anxiety

#### State Anxiety

The assumption of sphericity was not violated [χ^2^(5) = 8.699, *p* > 0.05] and there was a significant effect of time on state anxiety [*F*_(3, 267)_ = 3.071, partial η^2^ = 0.03]. Time point 4 had higher state anxiety scores (43.98 ± 5.23) than time point 1 (42.54 ± 4.71, *p* < 0.05) (see Table 2).

To determine if participants had relevant symptoms of state anxiety a cut-off score of 40 or higher was used (Addolorato et al., 1999; Julian, 2011). The highest rate of state anxiety symptoms in the sample population occurred in time point 2 (60%, 54/90). Time point 4 was the second highest at time point 4 with 59% (53/90) of the population experiencing symptoms of state anxiety. Time point 1 was the next highest at 58% (52/90). Time point 3 had the lowest rate of symptoms of anxiety with 52% (47/90) of the population scoring over 40.

#### Trait Anxiety

Sphericity was assumed [χ2(5) = 3.615, *p* > 0.05] and there was no significant effect of time on trait anxiety [*F*_(3, 267)_ = 2.135, *p* > 0.05, partial η^2^ = 0.02].

Trait anxiety was positively associated with loneliness at all four time points. Trait anxiety was positively associated with depressive symptoms at all four time points.

### Mood State

Mood states were broken down into their sub categories and repeated measures ANOVAS were calculated on the individual subcategories and TMD (see Table 2).

#### Anger

There was no effect of time on anger [*F*_(3, 267)_ = 1.912, *p* > 0.05, partial η^2^ = 0.02]. Sphericity was assumed [χ^2^(5) = 6.814, *p* > 0.05].

#### Vigor

There was a significant effect of time on vigor [*F*_(3, 267)_ = 13.346, *p* < 0.001, partial η^2^ = 0.13]. Time point 2 had the lowest vigor scores (10.71 ± 3.12) compared to time point 1 (12.4 ± 3.84, *p* < 0.001), time point 3 (12.32 ± 4.52, *p* < 0.001), and time point 4 (12.99 ± 4.16, *p* < 0.001). Sphericity was assumed [χ^2^(5) = 7.211, *p* > 0.05].

#### Fatigue

Using a Greenhouse-Geisser correction [χ^2^(5) = 22.90, *p* < 0.001], there was a significant effect of time on fatigue [*F*_(2.594, 230.870)_ = 3.865, *p* < 0.05, partial η^2^ = 0.04]. Feelings of fatigue lower at time point 4 (11.47 ± 5.07) when compared to time point 2 (13.08 ± 5.46, *p* < 0.05), and time point 3 (12.74 ± 5.18, *p* < 0.01).

#### Confusion

Using a Greenhouse-Geisser correction [χ^2^(5) = 18.382, *p* < 0.05], there was no significant effect of time on confusion [*F*_(2.658, 236.605)_ = 2.354, *p* < 0.08, partial η^2^ = 0.03].

#### TMD

There was a significant effect of time on TMD [*F*_(2.656, 236.413)_ = 5.987, *p* < 0.01, partial η^2^ = 0.06] when using a Greenhouse-Geisser correction [χ^2^(5) = 22.223, *p* < 0.001]. Time point 4 had the lowest TMD (35.92 ± 19.8) compared to time point 1 (41.05 ± 18.24, *p* < 0.05), time point 2 (43.26 ± 18.31, *p* < 0.01), and time point 3 (39.70 ± 19.71, *p* < 0.05).

### Physical Activity

#### Walking

There was no significant effect of time on walking [*F*_(2.132, 189.705)_ = 0.160, *p* > 0.05, partial η^2^ = 0.002] when using a Greenhouse-Geisser correction [χ^2^(5) = 56.570, *p* < 0.001].

#### Moderate

There was no significant effect of time on moderate intensity physical activity [*F*_(2.740, 243.852)_ = 0.791, *p* > 0.05, partial η^2^ = 0.009] when using a Greenhouse-Geisser correction [χ^2^(5) = 13.445, *p* < 0.05].

#### Vigorous

There was no significant effect of time on vigorous intensity physical activity [*F*_(2.566, 228.377)_ = 0.734, *p* > 0.05, partial η^2^ = 0.008] when using a Greenhouse-Geisser correction [χ^2^(5) = 23.445, *p* < 0.001].

#### Total Physical Activity

A Mauchly's Test of Sphericity was performed and the assumption of sphericity was violated [χ^2^(5) = 18.852, *p* < 0.01], therefore a Greenhouse-Geisser correction was used. There was no significant effect of time on total physical activity [*F*_(2.653, 236.099)_ = 0.594, *p* > 0.05, partial η^2^ = 0.007].

#### Physical Activity Participation

Time point 3 had the highest percentage of the population classified as participating in low/insufficient levels of physical activity (51%, 46/90). Forty-seven percent (47%; 42/90) of the sample population participated in low/insufficient levels of physical activity at time points 2 and 4. Time point 1 had 41% (37/90) classified with low/insufficient participation in physical activity.

Time point 1 had the highest participation in sufficient levels of physical activity participation with 59% (53/90) of the population. At time point 1, 54% (49/90) of individuals participated in moderate levels of physical activity, which was the highest proportion of any of the time points. During time point1, 4% (4/90) of participants engaged in high levels of physical activity. Fifty-three percent (53%, 48/90) of participants were classified as participating in sufficient levels of physical activity during time points 2 and 4. During time point 2, 49% (44/90) were in the moderate physical activity category and 4% (4/90) were in the high category. Forty-eight percent (48%, 43/90) were classified in the moderate category and 5% (5/90) were in the high category during time point 4. Time point 3 had the lowest proportion of the population classified as participating in sufficient levels of physical activity (48%, 44/90). At this time point, 41% (37/90) of individuals were in the moderate category and 8% (7/90) were classified in the high category.

### Pearson's *r*-Correlations

Loneliness was positively correlated to depression at time point 1 (*r* = 0.72, *p* < 0.01), time point 2 (*r* = 0.29, *p* < 0.05), time point 3 (*r* = 0.59, *p* < 0.01), and time point 4 (*r* = 0.64, *p* < 0.01). In addition, loneliness was positively correlated to anger at time point 1 (*r* = 0.31, *p* < 0.05), time point 2 (*r* = 0.52, *p* < 0.05), time point 3 (*r* = 0.38, *p* < 0.01), and time point 4 (*r* = 0.55, *p* < 0.01). Loneliness was positively related to fatigue at time point 1 (*r* = 0.41, *p* < 0.01), time point 2 (*r* = 0.55, *p* < 0.01), time point 3 (*r* = 0.42, *p* < 0.01), and time point 4 (*r* = 0.45, *p* < 0.01). Also, loneliness was positively correlated to TMD at time point 1 (*r* = 0.53, *p* < 0.01), time point 2 (*r* = 0.54, *p* < 0.05), time point 3 (*r* = 0.50, *p* < 0.01), and time point 4 (*r* = 0.61, *p* < 0.01). In contrast, loneliness was negatively associated to vigor at time point 1 (*r* = −0.32, *p* < 0.05), time point 3 (*r* = −0.40, *p* < 0.01), and time point 4 (*r* = −0.44, *p* < 0.01).

Depression scores were positively correlated with fatigue (see Table 3) at time point 1 (*r* = 0.62, *p* < 0.01), time point 2 (*r* = 0.41, *p* < 0.05), time point 3 (*r* = 0.71, *p* < 0.01), and time point 4 (*r* = 0.66, *p* < 0.01). In addition, depression scores were positively associated with TMD at time point 1 (*r* = 0.75, *p* < 0.01), time point 2 (*r* = 0.42, *p* < 0.01), time point 3 (*r* = 0.83, *p* < 0.01), and time point 4 (r = 0.86, *p* < 0.01). In contrast, depression scores were negatively associated with vigor at time point 1 (*r* = −0.48, *p* < 0.01), time point 3 (*r* = −0.51, *p* < 0.01), and time point 4 (*r* = −0.63, *p* < 0.01).

**Table 3:** Pearson's r Correlations examining the relationships between mental health outcomes and physical activity amount over four time points.

****	**Time Point 1**				**Time Point 2**				**Time Point 3**				**Time Point 4**			
	**Walk**	**Mod**	**Vig**	**Total**	**Walk**	**Mod**	**Vig**	**Total**	**Walk**	**Mod**	**Vig**	**Total**	**Walk**	**Mod**	**Vig**	**Total**
Lonely	−0.23^*^	−0.04	−0.11	0.15	−0.07	−0.11	−0.28^*^	−0.18	−0.09	−0.09	−0.16	−0.13	−0.02	0.03	0.20	−0.06
Depression	−0.31^*^	−0.06	−0.09	−0.19	−0.06	0.05	−0.17	−0.05	−0.23^*^	−0.21^*^	−0.13	−0.25^*^	−0.16	−0.12	−0.26^*^	−0.24^*^
State anxiety	0.22^*^	0.16	0.02	0.19	0.10	0.08	0.10	0.12	0.10	0.15	0.01	0.12	0.11	0.11	0.15	0.16
Trait anxiety	−0.07	−0.06	−0.25^*^	−0.13	−0.05	0.03	−0.07	−0.03	−0.16	−0.14	−0.09	−0.17	0.04	−0.05	−0.03	−0.01
Anger	−0.19	−0.01	−0.13	−0.12	−0.24^*^	−0.19	−0.29^*^	−0.30^*^	−0.20	−0.18	−0.07	−0.20	−0.11	−0.05	−0.10	−0.12
Vigor	0.39^**^	0.09	0.23^*^	0.28^*^	0.24^*^	0.30^*^	0.39^**^	0.40^**^	0.23^*^	0.24^*^	0.11	0.26^*^	0.18	0.18	0.29^**^	0.27^*^
Fatigue	−0.26^*^	−0.05	−0.16	−0.18	−0.12	−0.12	−0.28^*^	−0.20	−0.20	−0.16	−0.04	−0.18	0.10	−0.08	−0.12	−0.07
Confusion	−0.01	−0.13	0.1	0.06	−0.04	0.05	−0.08	−0.01	−0.04	−0.05	0.08	−0.02	0.05	0.07	0.03	0.07
TMD	−0.32^*^	−0.11	−0.18	−0.24^*^	−0.14	−0.20	−0.33^*^	−0.28^*^	−0.22^*^	−0.25^*^	−0.11	−0.25^*^	−0.09	−0.10	−0.17	−0.14

* *p < 0.05*;

** *p < 0.01; TMD, total mood disturbance*.

State anxiety was positively associated with vigor (see Table 3) at time point 1 (*r* = 0.29, *p* < 0.05), time point 2 (*r* = .29, *p* < 0.05), time point 3 (*r* = 0.37, *p* < 0.01), and time point 4 (*r* = 0.40, *p* < 0.01).

At time point 1, TMD was negatively associated with total physical activity (*r* = −0.24, *p* < 0.05). Anger (*r* = −0.30, *p* < 0.05) and TMD (*r* = −0.28, *p* < 0.05) were negatively associated with total physical activity at time point 2 (see Table 3). During time point 3, depression scores (*r* = −0.25, *p* < 0.05) and TMD (*r* = −0.25, *p* < 0.05) were negatively related to total physical activity (see Table 3). Depressionscores were negatively related to total physical activity (*r* = −0.24, *p* < 0.05) at time point 4. Vigor was positively associated with total physical activity at time point 1 (*r* = 0.28, *p* < 0.05), time point 2 (*r* = 0.40, *p* < 0.01), time point 3 (*r* = 0.26, *p* < 0.05), and time point 4 (*r* = 0.27, *p* < 0.05).

Loneliness (*r* = -.023, *p* < 0.05), depression scores (*r* = −0.31, *p* < 0.05), fatigue (*r* = −0.26, *p* < 0.05), and TMD (*r* = −0.32, *p* < 0.05) were negatively associated with walking during time point 1 (see Table 3). At time point 2, anger was negatively related to walking (*r* = −0.024, *p* < 0.05). Depression (*r* = −0.23, *p* < 0.05) and TMD (*r* = −0.22, *p* < 0.05) were negatively associated with walking at time point 3. In addition, vigor was positively associated with walking at time point 1 (*r* = 0.39, *p* < 0.01), time point 2 (*r* = 0.23, *p* < 0.05), and time point 3 (*r* = 0.23, *p* < 0.05). State anxiety was positively associated with walking (*r* = 0.22, *p* < 0.05) at time point 1.

During time point 3, depression scores (*r* = −0.21, *p* < 0.05) and TMD (*r* = −0.25, *p* < 0.05) were negatively associated with moderate intensity physical activity. Vigor was positively associated with moderate intensity physical activity during time point 2 (*r* = −0.30, *p* < 0.05) and time point 3 (*r* = 0.30, *p* < 0.05).

Participation in vigorous intensity physical activity was negatively associated with trait anxiety (*r* = -.025, *p* < 0.05) at time point 1. During time point 2, loneliness (*r* = −0.28, *p* < 0.05), anger (*r* = −0.29, *p* < 0.05), fatigue (*r* = −0.28, *p* < 0.05), and TMD (*r* = −0.33, *p* < 0.05) were all negatively associated with vigorous intensity physical activity. Depression scores were was negatively associated with participation in vigorous intensity physical activity (*r* = −0.26, *p* < 0.05) at time point 4. Vigor was positively associated with participation in physical activity at time point 1 (*r* = 0.23, *p* < 0.05), time point 2 (*r* = 0.39, *p* < 0.01), and time point 4 (*r* = 0.29, *p* < 0.01).

## Discussion

The primary purpose of this study was to identify any longitudinal changes in mental health outcomes and self-reported physical activity while social distancing practices were in place during the COVID-19 pandemic. The current study examined mental health and physical activity for 8 weeks (four time points) following the implementation of “safer at home” orders in Oklahoma. While there were no changes in physical activity over the 8 week data collection period, the COVID-19 pandemic and the safer at home orders had a major impact on mental health. It appeared that mental health declined during time point 2 regarding depression and state anxiety scores. Symptoms of depression and anxiety were much higher than compared to previous epidemiological data before the COVID-19 pandemic.

Data for time point one were collected three weeks after Oklahoma declared a state of emergency on March 16th and two weeks after “safer at home” orders were announced on March 24th which were initially effective until April 30 (Executive Department Amended Executive Order, 2020-07). Majority of COVID-19 cases in the following weeks, in which time point two data were collected, were in Oklahoma county and Cleveland county (adjacent counties); both of which house University of Oklahoma campuses (Health OSDo, 2020). On April 15, safer at home order was extended until May 6 and during this time frame, recollection data for time point three was collected (Governor Stitt Provides Update on State's Response to COVID-19, 2020). Phase one of reopening had begun when time point four data was collected (Reports, 2020). It is important to address what restrictions and policies were in place during the different time points that we collected, so that we can see over time what could also be affecting physical activity levels and psychological wellbeing in our sample population.

There were no significant changes in loneliness over the four time points. It has been shown that social isolation increases feelings of loneliness (Peplau, 1982). Longitudinal loneliness data, that was also collected during the COVID-19 pandemic, found that mean loneliness scores increased significantly over three monthly assessments (Killgore et al., 2020). We collected data for when lockdown began and when restrictions began to lift (6 week period) which could have been why we did not see the change over time. Despite loneliness level not changing over the four time points, mean loneliness scores at all time points in our study were the same or higher than other studies of similar design. Data examining individuals under a stay-at-home order have shown to be at an increased risk of experiencing feelings of loneliness (Tull et al., 2020). Older individuals are more likely to be socially isolated (Iliffe et al., 2007) and experience feelings of loneliness (Theeke, 2009). A study that examined loneliness and health-related behaviors in 8,688 older adults that live alone, found the average UCLA loneliness score to be 4.2 ± 1.4 in the tested population compared to our 17.6 ± 15.8–20.4 ± 14.5 ranges over the four time points (Shankar et al., 2011). Another study that examined the relationship between coronavirus anxiety and loneliness among college students had a lower mean loneliness score (14.4 ± 4.65) compared to our population (Arslan et al., 2020). Individuals who suffer from loneliness are more likely to experience depression (Matthews et al., 2016), anxiety (Cacioppo et al., 2006; Arslan et al., 2020), and negative mood (Loucks, 1980).

Depression scores were highest during time point two compared to the other three time points, and there were differences between time point one and four. Time point four revealed that 42% of participants had depressive symptoms, whereas time point two indicated that 100% of the sample population met the depression cut off score of 16 on the CES-D (Brummett et al., 2003). All four time points had significantly higher rates of feelings of depressive symptoms compared to rates before COVID-19. The Centers for Disease Control and Prevention (CDC) reported that 8.1% of adults in the US over the age of 20 had depressive symptoms over a 2-week period (Brody et al., 2018). A study that examined social isolation during the SARS-epidemic, found that 31.2% of participants had depressive symptoms (Hawryluck et al., 2004); our sample population had a higher mean percentage at all time points. More recent literature, conducted during COVID-19 has reported elevated feelings of depression. One study found a three-fold increase in depressive symptomology in participants during COVID-19 compared to pre-COVID-19 rates (Ettman et al., 2020). As mentioned previously, loneliness is positively correlated to depression and it is plausible that loneliness is a culprit to elevated depressive symptoms during the time points in which data were collected. Furthermore, depressive symptoms can stem from stressful events, such as loss of a loved one and economic difficulties. With death tolls increasing (Gallagher et al., 2020; Yildirim and Güler, 2020) and growing concerns about an economic recession caused by COVID-19 (Hertz-Palmor et al., 2020; Wilson et al., 2020), these events have been shown to be contributors to increased depression rates and its associated symptoms (Gallagher et al., 2020; Hertz-Palmor et al., 2020; Wilson et al., 2020; Yildirim and Güler, 2020). During time point 4, the number of people with depressive symptoms was reduced compared to the other time points, and with the start of phase one occurring at this time point, some economic stress may have be alleviated as some people may have been returning to work.

While there were no differences in trait anxiety, state anxiety changed over the 6 week data collection period. Trait anxiety has been shown to be a stable metric over time and is not sensitive to short term changes in situation (Skapinakis, 2014). A cut-off score of 40 or higher was used to determine symptoms of state anxiety (Addolorato et al., 1999; Julian, 2011) and it was found that at all time points more than half of the population had symptoms of state anxiety. The current study found higher rates for symptoms of anxiety at all time points compared to nationwide statistics pre-COVID-19. The CDC reported that 15.6% of adult Americans experienced at least mild symptoms of anxiety during 2019 (Terlizzi, 2019). As time went on, mean anxiety scores and total number of individuals with anxiety-related symptoms increased, with time point four having higher anxiety scores than time point one. We can speculate that this could have been due to the state of Oklahoma reopening despite increases in COVID-19 cases and deaths. When we first collected data for time point one (03/30/2020–4/13/2020) there were 1,327 cases and 51 deaths reported on 04/06/2020 (half way point for time point one data collection period) and at time point four (4/27/2020–05/11/2020) there were 4,044 cases and 283 deaths reported on 05/04/2020. This was an increase of 205% for total COVID-19 cases in the state of Oklahoma and 455% increase for total number of deaths. It has been speculated that “coronaphobia,” a termed coined by Asmundson and Taylor (Asmundson and Taylor, 2020), has led to increases in anxiety because individuals who are anxious about COVID-19 tend to experience a coherent set of mental health and mood disturbances that are triggered by thoughts, information, or news associated with the virus (Evren et al., 2020). Similarly to depressive symptoms, symptoms of anxiety has been positively associated with COVID-19-related stressors including financial worries (Hertz-Palmor et al., 2020; Wilson et al., 2020), death-related anxiety (Gallagher et al., 2020; Yildirim and Güler, 2020), disruptions to daily life including academic disruptions (Cao et al., 2020), and ambiguity of when COVID-19 would end (Freeston et al., 2020).

The COVID-19 pandemic has changed the usual life of individuals across the globe which has impacted the many facets of mood. Total mood disturbance was lowest at time point four compared to the other three time points. Two of the most altered mood states were vigor and fatigue. There were no changes over time in anger, or confusion. Vigor was highest at time point one and lowest at time point two. Fatigue was lowest at time point four compared with time point two and three. By monitoring the effect of COVID-19 has on mood, we can see how well society is dealing with the COVID-19-related societal restrictions. Using a weekly assessment of mood, The YouGov website in the United Kingdom found that the nation reporting's of feeling “happy” had dropped from 50% in March 2020 to 26% a month later, “scared” had risen from 11 to 34%, “bored” increased from 19 to 34%, and “stressed” went up from 41 to 48% demonstrating that the collective mood of the nation had been altered as a result of lockdown restrictions (YouGov., 2020). A review of 24 studies that examined the psychological effects of social isolation and being in quarantine found that PTSD, confusion, and anger were some of the negative associated effects with fear, frustration, and boredom being the major stressors that may contribute to mental health issues (Brooks et al., 2020). Overall life satisfaction has been shown to decrease due to reductions in social participation (Ammar et al., 2020). Mood disturbance in total mood and with fatigue and vigor especially, may also be explained by reduced physical activity levels and increased sedentary behaviors during safer at home orders in Oklahoma.

The anti-depressant effect of exercise has been well established (Dunn et al., 2005; Siqueira et al., 2016) Exercise has also been prescribed as a treatment for mood disorders (Hearing et al., 2016). While our sample population demonstrated fluctuations in depressive symptoms and mood, mean physical activity amount did not change across the 8 week data collection period in walking, moderate, and vigorous physical activity. However, the total number of people that met sufficient levels of physical activity decreased during time point two and three before rebounding back up at time point four. At time point 1 more people participated in adequate amounts of physical activity suggesting that individuals used additional free time at home to participate in increased physical activity, either intentionally or unintentionally as household chores, however, as time went on, the total number of people that met sufficient physical activity dropped until time point four. Loneliness, depressive symptoms, fatigue, and TMD were negatively associated with walking at one or more time points. Interestingly, during time point two where mood had the greater disturbances and depressive symptoms were notably higher; loneliness, anger, fatigue, and TMD, were all negatively associated with vigorous intensity physical activity but only anger was associated with walking intensity. This may indicate that more intense physical activity rather than walking during COVID-19 restrictions may be more appropriate for positive mental health outcomes. This is consistent with findings from Currier et al. (2020) where higher intensity physical activity was associated with reduced prevalence of depression in men. Furthermore, a negative association was found between moderate-vigorous physical activity and poor mental health in both males and females (Jacob et al., 2020) further suggesting that participating in higher intensity physical activity during self-isolation is associated with better mental health outcomes.

Our study did have several limitations. Due to the almost instantaneous decision to implement safer at home orders, it was not possible to collect pre-social distancing measures, however we made every effort to compare our data to previous epidemiological data. It was decided not to ask our participants to recollect how they felt and how much they exercised prior to safer at home policies due to recall bias. However, all measurements taken at all time points required self-report of feelings, and physical activity amounts from the previous week leading up to the appropriate time point intended on being measured. Secondly, we did not have a control group as the majority of the world was undergoing some kind of social-distancing practice simultaneously. Although not intended, the majority of our sample was OU personnel and affiliates. We recruited our sample population from OU Mass Mail, social media posts, and snowballing family and friend recruitment of people who had completed the questionnaire. Because of this, generalizations made to the whole population should be approached with caution as university mass mail led to a larger presence of young adults in the sample. Additionally, in our female participants, we did not control for menstrual cycle and other cyclical hormonal changes and it has been previously found that mood is influenced by menstrual cycle phase (Moos et al., 1969). Finally, we did not control for pre-existing mental health conditions which may have influenced our results as the shelter at home, and social distancing guidelines may have exacerbated some pre-existing mental health concerns. People with anxiety-related or mood disorders have been shown to be more negatively affected by COVID-19 compared to those with no mental health disorder (Asmundson et al., 2020).

## Conclusion

Social isolation and social distancing practices has had a negative effect on symptoms of depression, anxiety symptoms, and mood over time. While physical activity and loneliness remained unchanged over the four time points, less people participated in the recommended physical activity guidelines and loneliness levels were higher than other studies. It appeared that depressive symptoms and total mood disturbance was elevated during time point two and state anxiety scores were highest at time point four. Depressive symptoms were much higher than average compared to previous epidemiological data. This study supports the previously made connection between mental health and physical activity.

Further research should address the long-term effects of social isolation, and social distancing on mental health and whether reduced physical activity due to these social restrictions is going to have long-term implications on both mental and physical health. Additionally, research examining the dose-response of physical activity during social isolation and impacts on mental health should be addressed. Stay at home orders has had a definitive impact on the psychological wellbeing of individuals, globally. Those who are vulnerable to negative mental health outcomes, especially individuals who are facing greater stress and ambiguity should be given access to affordable resources and health care. Local governments and policy makers should encourage physical activity and provide appropriate guidelines that ensure safe social-distancing practices while partaking in physical activity that will benefit both physical and mental wellbeing. Ongoing evaluation examining the effects of stay at home orders on mental health and health behaviors such as reduced physical activity is warranted to help govern guidelines and policy regarding both mental and physical health by creating strategies to promote physical activity and reduce sedentary behaviors.

## Data Availability Statement

The raw data supporting the conclusions of this article will be made available by the authors, without undue reservation.

## Ethics Statement

The studies involving human participants were reviewed and approved by University of Oklahoma ethics committee. The patients/participants provided their written informed consent to participate in this study.

## Author Contributions

JP and GC were involved in the conceptualization. JP collected data and data analysis. GC and DL worked on statistical analysis and results. JP wrote manuscript. GC wrote abstract. RL and CB offered mentorship, guidance, and edits. All authors contributed to the article and approved the submitted version.

## Conflict of Interest

The authors declare that the research was conducted in the absence of any commercial or financial relationships that could be construed as a potential conflict of interest.
